# Protective Responses Induced by Chiral 3-Dichloroacetyl Oxazolidine Safeners in Maize (*Zea mays L.*) and the Detoxification Mechanism

**DOI:** 10.3390/molecules24173060

**Published:** 2019-08-22

**Authors:** Shuang Gao, Yan-Yan Liu, Jing-Yu Jiang, Ying Fu, Li-Xia Zhao, Chun-Yan Li, Fei Ye

**Affiliations:** 1 College of Science, Northeast Agricultural University, Harbin 150030, China; 2 College of Resources and Environment, Northeast Agricultural University, Harbin 150030, China

**Keywords:** chiral safener, biological activity, antioxidative enzyme activity, protection mechanism, chlorsulfuron

## Abstract

Herbicide safeners selectively protect crops from herbicide injury while maintaining the herbicidal effect on the target weed. To some extent, the detoxification of herbicides is related to the effect of herbicide safeners on the level and activity of herbicide target enzymes. In this work, the expression of the detoxifying enzyme glutathione S-transferase (GST) and antioxidant enzyme activities in maize seedlings were studied in the presence of three potential herbicide safeners: 3-dichloroacetyl oxazolidine and its two optical isomers. Further, the protective effect of chiral herbicide safeners on detoxifying chlorsulfuron in maize was evaluated. All safeners increased the expression levels of herbicide detoxifying enzymes, including GST, catalase (CAT), and peroxidase (POD) to reduce sulfonylurea herbicide phytotoxicity in maize seedlings. Our results indicate that the *R*-isomer of 3-(dichloroacetyl)-2,2,5-trimethyl-1,3-oxazolidine can induce glutathione (GSH) production, GST activity, and the ability of GST to react with the substrate 1-chloro-2,4-dinitrobenzene (CDNB) in maize, meaning that the *R*-isomer can protect maize from damage by chlorsulfuron. Information about antioxidative enzyme activity was obtained to determine the role of chiral safeners in overcoming the oxidative stress in maize attributed to herbicides. The interaction of safeners and active target sites of acetolactate synthase (ALS) was demonstrated by molecular docking modeling, which indicated that both isomers could form a good interaction with ALS. Our findings suggest that the detoxification mechanism of chiral safeners might involve the induction of the activity of herbicide detoxifying enzymes as well as the completion of the target active site between the safener and chlorsulfuron.

## 1. Introduction

Sulfonylurea herbicides are widely applied in agriculture, as they are highly effective. They can block the biosynthesis of leucine, isoleucine, and valine, which are essential branched-chain amino acids in plants, by inhibiting the activity of acetolactate synthase (ALS). Presently, about 30 varieties of sulfonylurea herbicides have been commercialized, and they are suitable for the production of all major agronomic crops, such as wheat, rice, corn, soybean, rape, sugar beet, sugar cane, and lawn. They exhibit excellent herbicidal effects on a broad range of weeds. Nevertheless, the phytotoxicity of sulfonylurea herbicides on nontarget crops has been reported [1,2]. According to a survey, after the application of chlorsulfuron, crop damage and yield decline can occur [3]. The damage caused by the excessive use of pesticides has attracted the attention of researchers [4].

Herbicide safeners have proved to be effective at protecting crops from herbicide injury while maintaining the herbicidal effect on the target weed species [5,6]. To some extent, the detoxification of herbicides is related to the effect of herbicide safeners on the level and activity of herbicide target enzymes [7]. The safeners isoxadifen-ethyl and mefenpyr-diethyl were shown to improve the tolerance of crops to sulfonylurea herbicides by inducing cellular xenobiotic detoxification mechanisms [8]. Three potential herbicide safeners have been reported to enhance the expression of ALS and glutathione S-transferase (GST), thereby weakening the likelihood of maize injury caused by chlorimuron-ethyl [9]. The literature indicates that the content of glutathione (GSH) and the activity of GST in crops increase with the application of safeners. The binding of GSH and herbicides through nucleophilic substitution reactions is catalyzed by GST, and the formation of products without active chemical properties is considered to be a possible detoxification mechanism [10,11]. The application of herbicides significantly increases the probability of oxidative damage. Plants protect themselves from oxidative injury introduced by herbicides by adjusting physiological and metabolic processes. One important approach is to enhance the activity of antioxidases, including GST, catalase (CAT), superoxide dismutase (SOD), and peroxidase (POD) [12,13]. According to the literature [14,15], CAT activity in plants increases during herbicide exposure, which is considered to be a response to oxidative stress caused by herbicides. Similarly, POD and GST also play roles as indicators of oxidative stress [16,17,18]. 

In recent years, molecular docking, which can provide valuable information on molecular interactions, has been used to explore reasonable bonding methods as well as to develop new herbicides and safeners. Based on molecular docking, a series of *p*-hydroxyphenylpyruvate dioxygenase (HPPD) inhibitors have been designed and synthesized, and the synthesized compounds were shown to be potential candidates for novel herbicides [19,20]. Anilides were synthesized through simple methods by Sartori et al. [21], and the herbicidal activities of the anilides were evaluated. An in silico study was performed on anilides with herbicidal activity, and histone deacetylase was suggested to be the target enzyme for the activity of anilides in plants. This was helpful for developing a new approach to control weeds. Yan et al. reported a novel aryloxyphenoxypropionate herbicide based on benzofuran and investigated its possible herbicidal mechanism by molecular docking [22]. The molecular docking experiment performed by Ye et al. [11,23] suggested that the reason that the safener had a protective effect might be due to competition between the safener and the herbicide in the target enzyme active pocket.

3-Chloroacetyl oxazolidine derivatives have been shown to protect crops from injury caused by herbicides [24]. Some known 3-dichloroacetyl substituted oxazolidines are chiral compounds, and chirality might have a certain effect on biological activity [25,26]. Chiral R-29148 and 3-dichloroacetyl substituted oxazolidines were successfully synthesized in our previous report [27]. Therefore, the present study aimed to ascertain the mechanism by which the raceme R-29148 and its chiral isomers protect maize from injury caused by chlorsulfuron and verify the supposition that herbicide detoxification can be enhanced by safeners. The protective effects of chiral safeners related to maize seedling growth; GSH content; and GST, POD, and CAT activity were examined to indicate the potential of chiral safeners to promote herbicidal metabolism on the expression of detoxifying and antioxidative enzyme activity. So far, few studies have been reported on the ability of chiral safeners to protect maize from injury caused by chlorsulfuron. The present study focused on the detoxification abilities of racemic R-29148, the *R*-isomer, and the *S*-isomer towards chlorsulfuron. The detoxification abilities of the safeners were decided by the degree of conjugation with glutathione in maize. On the basis of antioxidase activity, the role of chiral safeners in resisting oxidative stress induced by herbicides in maize was examined. Molecular docking was carried out to understand the detoxification mechanisms of chiral safeners.

## 2. Results

### 2.1. Results of Determination of the Growth Index

**Figure 1:**
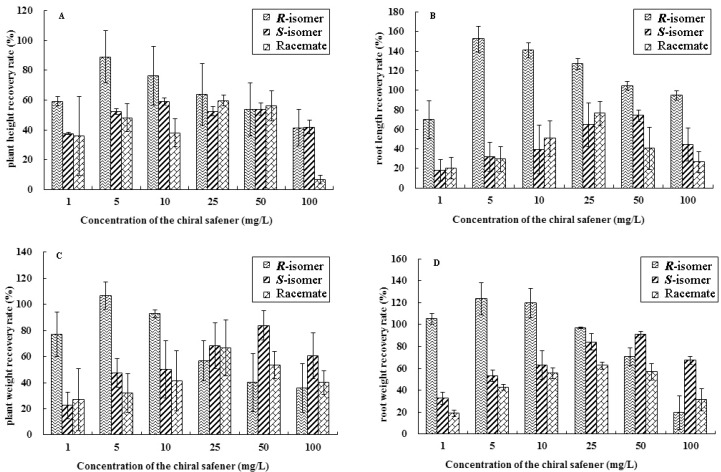
Recovery rates expressed as the growth level of maize treated with chlorsulfuron and safeners.

The growth index inhibition rate of maize treated by chlorsulfuron decreased significantly. To obtain suitable treatment conditions, safener solutions of different concentrations were evaluated for their ability to reduce injury caused by chlorsulfuron. The growth index recovery rates of maize are shown in Figure 1. The results show that all three chiral safeners could significantly reduce the inhibition caused by chlorsulfuron. The recovery rates of maize seedlings treated by the *R*-isomer and racemic R-29148 were higher than those treated by the *S*-isomer. According to Figure 1, the optimal protective effects of the three safeners were as follows: R-29148 with a concentration of 25 mg·L^−1^, *R*-isomer with a concentration of 5 mg·L^−1^, and *S*-isomer with a concentration of 50 mg·L^−1^. 

### 2.2. GSH and GST Results

#### 2.2.1. GSH Content

The efficiency of safeners for detoxification was found to be connected with the conjugation level of glutathione in maize [28]. In our evaluation, the increase in the GSH level in maize tissues treated with safener–chlorsulfuron was greater than those treated with chlorsulfuron (Table 1). Similarly, the GSH content in the seedling tissue treated by *R-*isomer–chlorsulfuron was greater than those treated by chlorsulfuron alone. After treatment with the *R*-isomer, the GSH content in the maize tissues increased noticeably. The results indicated a relationship between the enhanced GSH level in the maize seedlings and the protective activity of the herbicide safener. The safener-induced increase in GSH has also been reported previously [29].

#### 2.2.2. GST Activity

Safeners were shown to motivate the activity of GST, while toxicity was effectively reduced through GST-catalyzed conjugation of GSH to the herbicide. In contrast to the control, the activity of GST in maize treated by chlorsulfuron increased. The use of a safener and chlorsulfuron also led to an increase in the in vivo activity of GST (Table 2). When chlorsulfuron acted as the substrate instead of 1-chloro-2,4-dinitrobenzene (CDNB), the in vitro activity of GST also increased, which could be attributed to the chiral safeners (Table 2). The data indicate that different levels of protective efficiency of the safener on maize might derive from the different activity degrees of GST on CDNB or the chlorsulfuron substrate. In this study, all three safeners were shown to effectively induce the activity of GST, while the best efficiency was obtained with the attendance of the *R*-isomer.

#### 2.2.3. Kinetic Parameters of GST

The enzymatic extracts from maize roots were used to determine the kinetic parameters of GST in maize (Table 3). Table 3 indicates that *V*_max_ decreased while *K*_M_ showed the opposite trend when the maize was treated with chlorsulfuron. Compared with the control, *V*_max_ increased and *K*_M_ decreased after treatment with the *R*-isomer and racemic R-29148. It can be observed from Table 3 that the *R*-isomer obviously affected the induction and dynamics of GST activity. Other safeners also induced the affinity of GST to the substrate in the conjugated reaction. This is similar to the GSH content and GST activity results.

### 2.3. POD and CAT Activity

POD and CAT were shown to protect crops from the stress caused by herbicides because their activities are related to the reduction of oxidative stress induced by high-dose herbicides [30]. In order to evaluate the protective effectiveness of safeners, the influences of safeners and chlorsulfuron on the activity levels of POD and CAT were determined (Table 4). In the case of treatment with chlorsulfuron alone, an obvious increase in POD activity in the roots of maize was observed compared with the control. After being treated by the *R*-isomer, the POD activity decreased. This decrease in POD activity suggests that it might play an important role in the detoxification of herbicides under treatment with chiral safeners. 

CAT can also protect crops and reduce the stress caused by herbicides by participating in oxidative stress metabolism [14,15]. Compared with the control, the activity of CAT in maize treated with chlorsulfuron increased, while CAT activity in maize treated by the three safeners decreased, as shown in Table 4.

### 2.4. Molecular Docking Studies

**Figure 2:**
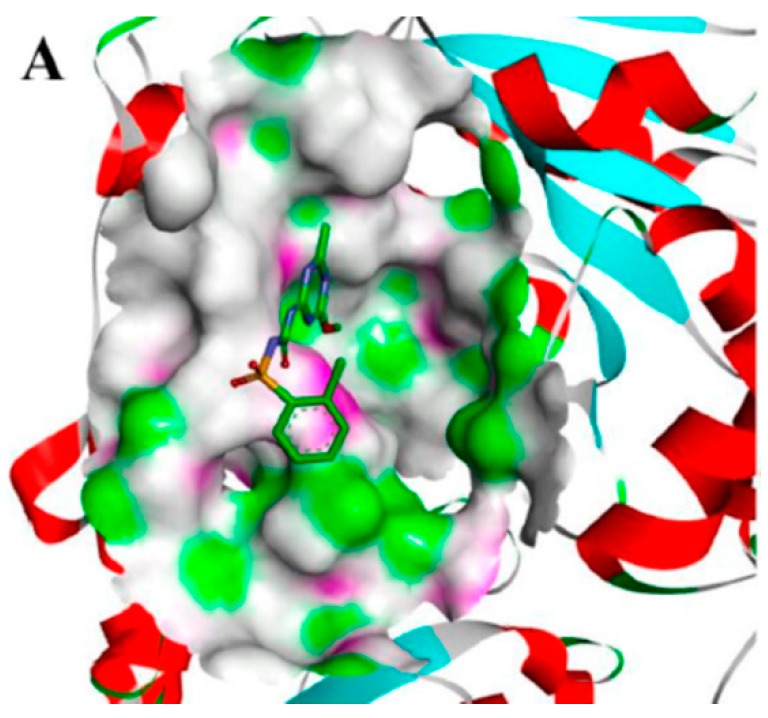
The docking model of chlorsulfuron with the acetolactate synthase (ALS) active site.

**Figure 3:**
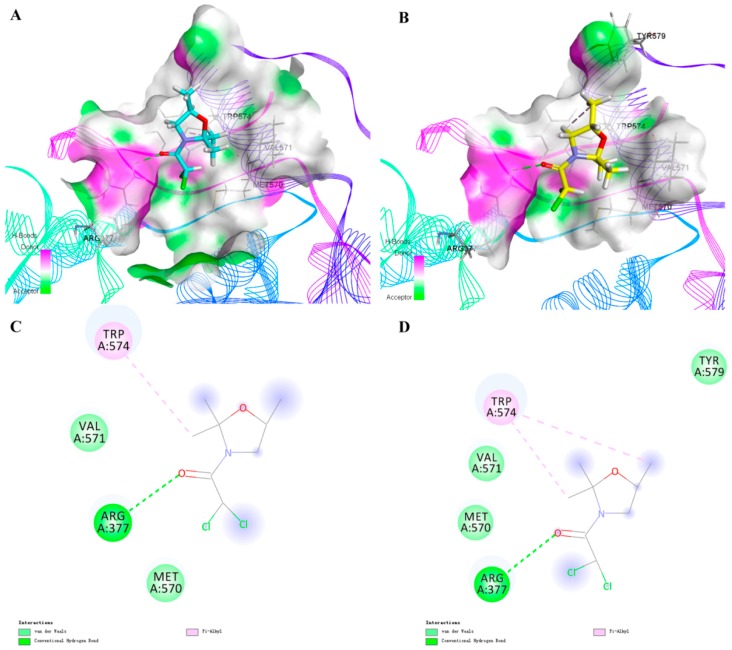
The docking model of (**A**) the *R*-isomer and (**B**) the *S*-isomer with the ALS active site, and the receptor–ligand interaction of (**C**) the *R*-isomer and (**D**) the *S*-isomer with the ALS active site.

**Figure 4:**
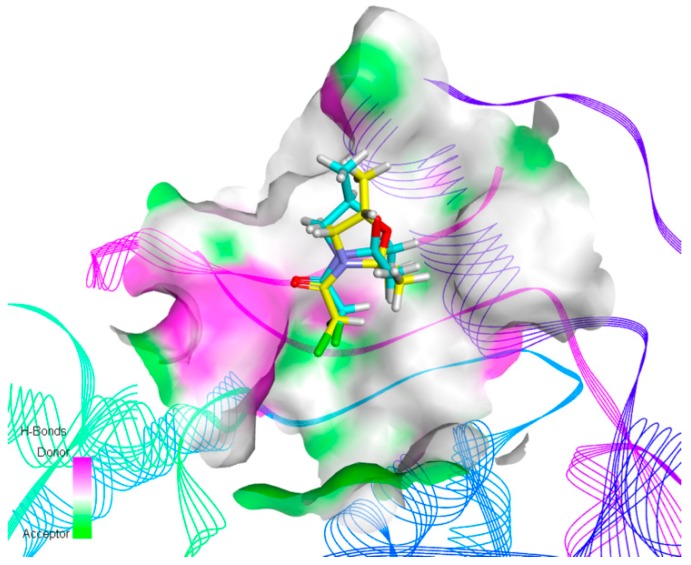
Overlay of the docking model of the two isomers with the ALS active site.

For the purpose of understanding the effects of chiral safeners on herbicide target enzymes, molecular docking experiments were carried out. Chlorsulfuron was docked with ALS, and the docking model is shown in Figure 2. Two approximately vertical portions of the entire molecule were observed to be located at the entrance of the channel and embedded in the channel, respectively. Thus, the active channel was blocked and the substrate was prevented from entering the active pocket channel. In comparison, the *R*-isomer and the *S*-isomer were used to dock with ALS to determine the detoxification mechanism (Figure 3). Figure 3A,B show the docking modeling of (A) the *R*-isomer and (B) the *S*-isomer with the ALS active site, respectively. Obviously, as shown in Figure 3A,B, the two isomers exhibited similar binding positions at the active site of ALS, which was also similar to chlorsulfuron. The scores of the *R*-isomer and *S*-isomer were similar: 3.43 and 3.25, respectively. The docking models of (A) the *R*-isomer and (B) the *S*-isomer with the ALS active site are shown in more detail in Figure 3C,D, respectively. A π–alkyl interaction with the amino acid TRP574, a hydrogen bond interaction with ARG377, and a van der Waals interaction with VAL571 and MET570 formed in the molecule of the *R*-isomer, while two π–alkyl interactions with the amino acid TRP574; a hydrogen bond interaction with ARG377; and van der Waals interactions with VAL571, MET570, and Tyr579 formed in the molecule of the *S*-isomer. The docking models of the two isomers with the ALS active site were overlaid, and the result shown in Figure 4 indicates that the methyl group at the chiral C in the oxazole ring of the *R*-isomer could not interact with Tyr579 and TRP574 due to the methyl group in the *R*-isomer being directed outward and thus away from the two amino acids. In contrast, the methyl group in the *S*-isomer configuration was directed inward, close to Tyr579 and TRP574, and could form a good interaction with the two amino acids. The results revealed by molecular docking proved that the detoxifying activity of the *R*-isomer was higher than that of the *S*-isomer, which is in accordance with the results obtained in the biological activity assay. The results indicate that the detoxification mechanism of chiral safeners might be attributed to the completion of the target active site between the *R*-isomer and chlorsulfuron, which hinders the action of herbicides at the active site of ALS, resulting in a loss of efficacy of chlorsulfuron.

## 3. Discussion

Glutathione conjugation has been reported to be responsible for herbicide resistance in plants [31]. GST is a key enzyme that can catalyze the conjugation of toxic electrophilic groups with reduced glutathione, so as to achieve the purpose of detoxification in plant cells. It can eliminate reactive oxygen substances by conjugating with glutathione and catalyze the reaction of the thiol group in reduced GSH with herbicides [32,33]. In this way, the toxicity of herbicides is detoxified [34]. This means that, to some extent, the effectiveness of safeners on detoxification could be indicated by the degree of conjugation of glutathione in maize. In summary, the *R*-isomer can improve the GSH content as well as the activity of GST in maize seedlings so as to promote the conjugation of glutathione with chlorsulfuron and then protect maize from injury caused by sulfonylurea herbicides. The GST activity toward CDNB was previously reported to increase significantly after treatment with a safener compared with treatment with chlorsulfuron [35]. Similarly, our results indicate that the *R*-isomer changed the kinetic parameters *V*_max_ and *K*_M_ obviously, while the other two safeners led to relatively small changes. Thus, GST could be induced to catalyze the conjugation of chlorsulfuron and GSH by chiral safeners. This process is considered to be responsible for the detoxification.

In this study, the effectiveness of the antioxidative system consisting of POD and CAT was investigated. POD and CAT are the primary enzymes that can scavenge H_2_O_2_ in plant cells, and they are considered to be related to the mechanism of herbicide-induced oxidative stress. The antioxidative system can protect crops from the stress induced by excess herbicide [36,37]. The literature indicates that the activities of POD and CAT increase in several plant species when exposed to herbicides [38,39,40]. Herbicides and safeners induce significant changes in both antioxidative enzyme activities and the antioxidative stress response. In this study, the activities of POD and CAT in maize decreased after treatment with R-29148 and the *R*-isomer, which indicated that the chiral safener showed a certain resistive effect against oxidative stress in maize. Treatment with the *S*-isomer reduced CAT activity significantly. However, no significant change in POD activity was recorded in maize treated with the *S*-isomer. The activities of the two enzymes decreased after treatment with the three safeners, in contrast to their responses to the herbicide. It could be concluded that the *R*-isomer performed the best at reducing oxidative stress caused by chlorsulfuron in maize, which is in accordance with the growth index shown in Figure 2. This mechanism could be an important approach to explain the detoxification of chiral safeners in maize.

The molecular docking experiment indicated that the chiral isomer combined with ALS to form a hydrogen bond, and the complex formed showed low overall energy. The safener competed with chlorsulfuron for the active site of ALS, hindering the activity of herbicides on the active site and resulting in a loss of efficacy of chlorsulfuron. Docking modeling is demonstrated in more detail in Figure 3C,D. Hydrogen bonds were the main bonds for binding the safener to the target site, including a π–alkyl interaction with the amino acid TRP574, a hydrogen bond interaction with ARG377, and van der Waals interactions with VAL571 and MET570. The combination mode of the safener at the ALS active site was similar to that shown in Figure 2, with the two approximately vertical portions of the molecule located at the entrance of the channel and embedded in the channel, respectively. The literature [41] indicates that ALS inhibitors could prevent substrates from entering the active pocket channel by plugging the entrance and that the herbicidal activity is related to the interaction of the inhibitor with the amino acid residues in the vicinity of the active site. The safener preemptively occupied the active site, resulting in the inability of chlorsulfuron to match the target site. In addition, the safener had a smaller structure than chlorsulfuron, with a heterocyclic ring containing O and N, and easily formed a bridge structure by hydrogen bonding. It could not block the entrance of the channel completely. There were still gaps that only allowed small molecules to pass, while macromolecules were blocked. Thus, small substrates could enter the channel and react with the active site. In this way, detoxification was achieved. However, excessive interaction might cause blockage of the entrance to the channel. In this study, the detoxifying activity of the *R*-isomer with relatively weak interactions was better than that of the *S*-isomer, which showed relatively strong interactions with the target active site.

## 4. Materials and Methods 

### 4.1. Materials and Chemical Reagents

**Figure 5:**
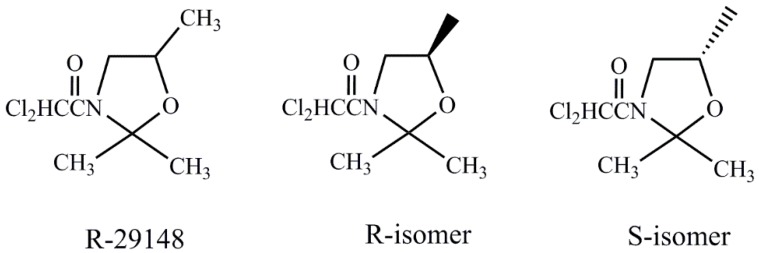
*R*-29148, *R*-isomer, and *S*-isomer employed in the test as potential safeners.

Chlorsulfuron wettable powder (20%) and standards were purchased from Liyang Chemistry Co., Ltd. and Aladdin Chemistry (Shanghai, China), respectively. 5, 5′-Dithiobis-(2-nitrobenzoic) acid (DTNB), CDNB, and GSH were provided by Sigma (Shanghai, China). Racemic R-29148, *R*-isomer, and *S*-isomer were synthesized in our laboratory with a purity of not less than 99.0% (Table 5, Figure 5). Detailed synthetic procedures, methods of purifications, and structure determination were described previously [27]. Other chemical solvents were obtained from Aladdin Chemistry (Shanghai, China). The maize seeds, named “Dongnong 253” (*Zea mays L.*), were germinated and raised in a growth chamber.

### 4.2. Plant Material and Growth Conditions

Before sowing, seeds were treated with safener solutions at six different concentrations (1, 5, 10, 25, 50, and 100 mg·L^−1^) at 26.5 °C for 12 h. The controls were soaked in distilled water instead of safener solution. Then, germinating was processed at 26.5 °C for 24 h and the seeds were sown in paper cups (8 × 12 cm) directly. Each cup contained 150 mL of quartz sand and six seeds. Quartz sand was washed with hydrochloric acid (10%, *v*/*v*) and then sterilized with sodium hypochlorite solution (5%, *w*/*v*). Sixty milliliters of chlorsulfuron solution (100 mg·L^−1^) were added to the sand quartz, and the water content in each cup was controlled at 13–15 g water per 100 g soil, which was 60–70% of the maximum water holding capacity. The seedlings were germinated and raised in the growth chamber with a relative humidity of 75% at 26.5 ± 1 °C and with a 12 h light photoperiod. 

The shoots and roots of maize under each treatment were collected and frozen with liquid nitrogen on the eighth day. Frozen samples were stored at −80 °C until further determination. Before the determination of enzyme activities (GSH, GST, POD, and CAT), frozen tissues were crushed in liquid nitrogen. The lengths and fresh weights (FWs) of the shoots and roots were also determined. All determinations were performed in triplicate. The recovery rate of the maize growth index was calculated to determine the optimal concentration of the safener. It was calculated by the following formula:

(1) $$\mathrm{Recovery}\;\mathrm{rate}\;(\%)\;=\;\frac{\mathrm{Treated}\;\mathrm{with}\;\mathrm{compounds}\;\mathrm{and}\;\mathrm{chlosulfuron}\;-\;\mathrm{Treated}\;\mathrm{with}\;\mathrm{chlorsulfron}}{\mathrm{Control}\;-\;\mathrm{Treated}\;\mathrm{with}\;\mathrm{chlorsulfuron}}.$$

### 4.3. GSH Content Assay

The GSH level was measured in accordance with Ismaiel and Papenbrock [42]. Homogenization was performed in sulfosalicylic acid (5%, *w*/*v*). The homogenate was centrifuged at 15,000× *g* for 20 min at 4 °C, and the supernatant was immediately used for the GSH content assay. The GSH content was determined spectrophotometrically at 412 nm with the DTNB reagent.

### 4.4. Extraction and Activity Determination of GST

The extraction and determination of GST activity was performed according to Del Buono and Ioli [31]. To measure the GST activity, 200 mg of frozen maize seedling tissue was ground into powder in liquid nitrogen and then homogenized in 1 mL of QB buffer (100 mM potassium phosphate buffer at pH 7.8 with 1 mM EDTA and polyvinylpyrrolidone at 5% *w*/*v*) at 4 °C. The homogenate was centrifuged at 15,000× *g* for 20 min at 4°C. The final assay mixture included 50 mM of phosphate buffer (pH 6.5), 1 mM of CDNB, 1 mM of GSH, and 0.5 mM of EDTA. The absorbance at 340 nm increased after the root extract was added for 180 s (60 s intervals). GST activity was defined as the amount of herbicide reaction with GSH catalyzed by 1 mg of GST in unit time (nmol·s^−1^·mg^−1^ protein). 

HPLC was used to determine the activity of GST in vitro against chlorsulfuron, in accordance with Scarponi et al. [7]. The GST extract was mixed with GSH and chlorsulfuron standard solution, and the reaction lasted for 2 h. Then, 10 μL of 3.6 M HCl was added into the reaction mixture to stop the reaction. The mixture was extracted by methanol and then collected to determine the concentration of chlorsulfuron by HPLC. The change in concentration of chlorsulfuron before and after the reaction was related to the GST activity, which was defined as the amount of chlorsulfuron reaction within 1 min with the unit mass of GST (nmol·min^−1^·mg^−1^ protein).

### 4.5. GST Kinetic Parameters Assay (CDNB)

The procedure reported by Del Buono et al. [43] was followed (with modifications) to evaluate the kinetic parameters of GST. Using double reciprocal plots, the kinetic parameters *V*_max_ and *K*_M_ were evaluated by linear regression analysis of 1/*V* versus 1/*S*. Different concentrations of CDNB (1.0–32.0 mM) were used to determine the GST activity, while the GSH concentration was 5 mM. 

### 4.6. CAT Activity Assay 

The total activity of CAT was evaluated by the decomposition rate of H_2_O_2_, which was determined at 240 nm in accordance with Hemanth et al. [44]. A total of 1.9 mL of H_2_O, 0.1 mL of enzyme extract, and 1 mL of hydrogen peroxide (0.3%, *v*/*v*) were mixed and incubated for 3 min, and then the absorbance at 240 nm was measured by a spectrophotometer. The CAT activity was defined as μmol H_2_O_2_ min^−1^·g^−1^ FW.

### 4.7. POD Enzyme Assay

To investigate the effect of the target safener, POD activity was determined based on a modified method from Obermeier et al. [30]. The mixture, which consisted of 1 mL of 50 mM sodium phosphate buffer (pH 7.0), 2 mL of 0.3% hydrogen peroxide, and 0.95 mL of 0.2% guaiacol was used for determination. After the addition of 0.01 mL of the enzyme extract to the reaction mixture, the mixture was determined spectrophotometrically at 470 nm for 5 min. The change in absorbance with the addition of H_2_O_2_ was recorded. The peroxidase activity was determined as mmol·min^−1^·g^−1^ FW.

### 4.8. Statistical Analysis 

All determinations were repeated three times. SPSS statistical software (version 16.0, International Business Machines Corporation, Armonk, NY, USA) was used for data analysis. Analysis of variance was performed on all data, while differences between treatments were evaluated by Duncan’s multiple range test. 

### 4.9. Molecular Docking Studies

The sketch module of the SYBYL-X 2.0 program package (Tripos, St. Louis, MO, USA) was used to build the 3D molecular structures of chlorsulfuron and the two isomers of 3-(dichloroacetyl)-2,2,5-trimethyl-1,3-oxazolidine. After the molecules were optimized, Gasteiger–Huckel charges were calculated. The crystal structure of ALS was obtained from the Protein Data Bank (PDB ID 1N0H). Accelrys Discovery Studio 2.5 provided the CDOCKER method for the docking modeling. The semiflexible docking method was performed in the docking studies and the most stable configuration of the two isomers was confirmed based on CDOCKER-ENERGY. Some cocrystallized small molecules and water were removed from the protein structure, and the protein was minimized using the CHARMM force field prior to docking. After the protein preparation, the active sites for the docking studies were defined within a range of 13.0 Å from the center of the known ligand [45]. The obtained ALS was used as the “Input Receptor”. Chlorsulfuron and the two isomers of 3-(dichloroacetyl)-2, 2, 5-trimethyl-1,3-oxazolidine were chosen as the “Input Ligands”.

During the docking process, the top 10 conformations were saved for each ligand based on the negative CDOCKER_ENERGY value after energy minimization. Finally, the default values were used in DS 2.5 if not mentioned otherwise.

## 5. Conclusions

On the basis of data obtained in this study, a conclusion could be made that the effects of racemic R-29148 and its chiral isomers on the growth and enzyme activity of maize could protect maize against injury from the sulfonylurea herbicide chlorsulfuron. The growth levels of maize and GST activity were significantly inhibited by chlorsulfuron, and this was tempered by adding the *R*-isomer. The data indicated that the safener could enhance the GST activity and then facilitate the resistance of maize seedlings to the phytotoxicity of chlorsulfuron. Based on the changes in POD and CAT activity, the *R*-isomer showed the best effect in detoxifying the plant from the effects of chlorsulfuron among the three safeners. Molecular docking modeling indicated that both isomers could form a good interaction with ALS. For the chiral pesticide, differences in the three-dimensional structure might lead to different activities, which was well reflected in this study. The different structures led to different interactions between safeners with the target active site, resulting in different detoxifying activities of the two isomers. It is suggested that the detoxification mechanism of chiral safeners might be attributed to the induction of the activity of herbicide detoxifying enzymes, as well as the completion of the target active site between the safeners and chlorsulfuron. Further studies should be carried out to explore the extra mechanism by which chiral safeners protect maize from injury induced by sulfonylurea herbicides.

## Figures and Tables

**Table 1:** Effects of safeners and chlorsulfuron on the glutathione (GSH) content in maize.

Treatment	GSH Content in Roots (μg·g^−1^)	GSH Content in Shoots (μg·g^−1^)
Control	3.775 ± 0.036 ^d^	7.841 ± 0.024 ^d^
Chlorsulfuron	4.862 ± 0.028 ^c^	15.164 ± 0.045 ^c^
*R*-isomer + Chlorsulfuron	7.068± 0.025 ^a^	26.662 ± 0.072 ^a^
*S*-isomer + Chlorsulfuron	6.339 ± 0.044 ^b^	21.767 ± 0.058 ^b^
R-29148 + Chlorsulfuron	6.155 ± 0.053 ^b^	20.053 ± 0.032 ^b^

Mean ± standard deviation. Values sharing same letters differ insignificantly (*p* > 0.05). The values correspond to the averages of three replicates.

**Table 2:** Effects of safeners and chlorsulfuron on the activity of glutathione S-transferase (GST) in maize.

Treatment	GST Activity In Vivo (μmol·min^−1^·mg^−1^ Protein)	Treatment	GST Activity In Vitro (nmol·min^−1^·mg^−1^ Protein)
Control	8.97 ± 0.30 ^d^	Control	123.73 ± 2.95 ^d^
Chlorsulfuron	10.43 ± 0.16 ^c^	Chlorsulfuron	-
*R*-isomer + Chlorsulfuron	19.44 ± 0.28 ^a^	*R*-isomer	195.67 ± 3.15 ^a^
*S*-isomer + Chlorsulfuron	12.19 ± 0.67 ^b^	*S*-isomer	162.43 ± 1.05 ^b^
R-29148 + Chlorsulfuron	11.84 ± 0.35 ^b^	R-29148	139.39 ± 1.88 ^c^

Mean ± standard deviation. Values sharing the same letters differ insignificantly (*p* > 0.05). The values correspond to the averages of three replicates.

**Table 3:** Effects of safeners and chlorsulfuron on the kinetic parameters of GST in maize.

Treatment	*V_max_* (nmol·min^−1^·mg^−1^ Protein)	*K_m_* (mmol·L^−1^)
Control	14.87 ± 0.30 ^c^	0.52 ± 0.04 ^a,b^
Chlorsulfuron	5.42 ± 0.44 ^d^	0.55 ± 0.15 ^a^
*R*-isomer	23.82 ± 0.23 ^a^	0.39 ± 0.05 ^c^
*S*-isomer	12.73 ± 0.67 ^d^	0.50 ± 0.12 ^a,b^
R-29148	17.54 ± 0.35 ^b^	0.48 ± 0.06 ^b^

Mean ± standard deviation. Values sharing the same letters differ insignificantly (*p* > 0.05). The values correspond to the averages of three replicates.

**Table 4:** Effects of safeners and chlorsulfuron on the peroxidase (POD) and catalase (CAT) activity of maize.

Treatment	CAT Activity (μmol·min^−1^·g^−1^ FW)	POD Activity (mmol·min^−1^·g^−1^ FW)
Control	2.09 ± 0.12 ^a^	1135 ± 2.05 ^b^
Chlorsulfuron	1.60 ± 0.06 ^b^	1387 ± 1.06 ^a^
*R*-isomer + Chlorsulfuron	1.04 ± 0.03 ^d^	863 ± 3.63 ^d^
*S*-isomer + Chlorsulfuron	1.20 ± 0.08 ^c^	1381 ± 2.35 ^a^
R-29148 + Chlorsulfuron	1.32 ± 0.10 ^c^	1047 ± 3.24 ^c^

Mean ± standard deviation. Values sharing the same letters differ insignificantly (*p* > 0.05). The values correspond to the averages of three replicates. Abbreviation: FW—fresh weight.

**Table 5:** Chemical names of tested safeners.

Safener	Chemical Name
R-29148	(R,S)-3-dichloroacetyl-2,2,5-trimethyl-1,3-oxazolidine
*R*-isomer	(R)- 3-dichloroacetyl-2,2,5-trimethyl-1,3-oxazolidine
*S*-isomer	(S)- 3-dichloroacetyl-2,2,5-trimethyl-1,3-oxazolidine
